# Vaccination coverage according to race or skin color in children born in 2017-2018 in Natal, Rio Grande do Norte, Brazil: a population survey

**DOI:** 10.1590/S2237-96222024v33e20231310.especial2.en

**Published:** 2024-12-13

**Authors:** Eliene Roberta Alves dos Santos, Isabelle Ribeiro Barbosa, José Cássio de Moraes, Ana Paula França, Carla Magda Allan Santos Domingues, Maria da Gloria Teixeira, Héllyda de Souza Bezerra, Nayre Beatriz Martiniano de Medeiros, Mayonara Fabíola Silva Araújo, Fábia Cheyenne Gomes de Morais Fernandes, Arthur Alexandrino, Ricardo Andrade Bezerra, ICV 2020 Group, Adriana Ilha da Silva, Alberto Novaes Ramos, Ana Paula França, Andrea de Nazaré Marvão Oliveira, Antonio Fernando Boing, Carla Magda Allan Santos Domingues, Consuelo Silva de Oliveira, Ethel Leonor Noia Maciel, Ione Aquemi Guibu, Isabelle Ribeiro Barbosa, Jaqueline Caracas Barbosa, Jaqueline Costa Lima, José Cássio de Moraes, Karin Regina Luhm, Karlla Antonieta Amorim Caetano, Luisa Helena de Oliveira Lima, Maria Bernadete de Cerqueira Antunes, Maria da Gloria Teixeira, Maria Denise de Castro Teixeira, Maria Fernanda de Sousa Oliveira Borges, Rejane Christine de Sousa Queiroz, Ricardo Queiroz Gurgel, Rita Barradas Barata, Roberta Nogueira Calandrini de Azevedo, Sandra Maria do Valle Leone de Oliveira, Sheila Araújo Teles, Silvana Granado Nogueira da Gama, Sotero Serrate Mengue, Taynãna César Simões, Valdir Nascimento, Wildo Navegantes de Araújo

**Affiliations:** 1 Universidade Federal do Rio Grande do Norte, Programa de Pós-Graduação em Saúde Coletiva, Santa Cruz, RN, Brazil; 2 Santa Casa de São Paulo, Faculdade de Ciências Médicas, São Paulo, SP, Brazil; 3 Organização Pan-Americana da Saúde, Brasília, DF, Brazil; 4 Universidade Federal da Bahia, Instituto de Saúde Coletiva, Salvador, BA, Brazil; 5 Universidade Federal do Rio Grande do Norte, Programa de Pós-Graduação em Saúde Coletiva, Natal, RN, Brazil; Universidade Federal do Espírito Santo, Vitória, ES, Brazil; Universidade Federal do Ceará, Departamento de Saúde Comunitária, Fortaleza, CE, Brazil; Faculdade Ciências Médicas Santa Casa de São Paulo, São Paulo, SP, Brazil; Secretaria de Estado da Saúde do Amapá, Macapá, AP, Brazil; Universidade Federal de Santa Catarina, SC, Brazil; Organização Pan-Americana da Saúde, Brasília, DF, Brazil; Instituto Evandro Chagas, Belém, PA, Brazil; Faculdade de Ciências Médicas Santa Casa de São Paulo, Departamento de Saúde Coletiva, São Paulo, SP, Brazil; Universidade Federal de Mato Grosso, Cuiabá, MT, Brazil; Universidade Federal do Paraná, Curitiba, PR, Brazil; Universidade Federal de Goiás, Goiânia, GO, Brazil; Universidade Federal do Piauí, Teresina, PI, Brazil; Universidade de Pernambuco, Faculdade de Ciências Médicas, Recife, PE, Brazil; Instituto de Saúde Coletiva, Universidade Federal da Bahia, Salvador, BA, Brazil; Secretaria de Estado da Saúde de Alagoas, Maceió, AL, Brazil; Universidade Federal do Acre, Rio Branco, AC, Brazil; Universidade Federal do Maranhão, Departamento de Saúde Pública, São Luís, MA, Brazil; Universidade Federal de Sergipe, Aracaju, SE, Brazil; Secretaria Municipal de Saúde, Boa Vista, RR, Brazil; Fundação Oswaldo Cruz, Mato Grosso do Sul, Campo Grande, MS, Brazil; Fundação Oswaldo Cruz, Escola Nacional de Saúde Pública Sergio Arouca, Rio de Janeiro, RJ, Brazil; Universidade Federal do Rio Grande do Sul, Porto Alegre, RS, Brazil; Fundação Oswaldo Cruz, Instituto de Pesquisa René Rachou, Belo Horizonte, MG, Brazil; Secretaria de Desenvolvimento Ambiental de Rondônia, Porto Velho, RO, Brazil; Universidade de Brasília, Brasília, DF, Brazil

**Keywords:** Cobertura Vacunal, Disparidades Socioeconómicas en Salud, Acceso a Servicios de Salud, Salud de las Minorías Étnicas, Los Determinantes Sociales de la Salud, Encuestas Epidemiológicas., Vaccination Coverage, Socioeconomic Disparities in Health, Access to Health Services, Health of Ethnic Minorities, Social Determinants of Health, Health Surveys

## Abstract

**Objective:**

To analyze vaccination coverage up to 24 months of age according to race/ skin color in the 2017-2018 live birth cohort in Natal, Rio Grande do Norte, Brazil.

**Methods:**

Population-based survey conducted in 2020 and 2021. Vaccination coverage up to 24 months of age was estimated according to administered, valid and timely doses. Crude association of race/skin color was estimated by calculating the crude Prevalence Ratio and respective 95% Confidence Intervals, using Poisson regression.

**Results:**

Of the 688 children in the selected cohort, there was greater coverage among Black children for administered doses (White 30.5%; Black 47.8%; 95%CI) and valid doses (White 25.8%; Black 40.1%; 95%CI), although without statistical significance, and lower coverage for timely doses, in the full schedule excluding yellow fever (PR = 0.21; 95%CI 0.04;0.90).

**Conclusion:**

There was lower timely coverage among Black children compared to White children.

## Study contributions

**Main results:**

It was found that racial inequalities and social disparities were reflected in vaccination coverage of Black children in the state of Rio Grande do Norte, showing lower prevalence of timely and full vaccination schedules among those children.

**Implications for services:**

The results demonstrate the need to strengthen equitable public policies and the implementation of practices that seek to improve vaccination coverage, thus reducing racial inequalities in child immunization.

**Perspectives:**

Health service managers will be able to plan actions and strategies in childhood immunization services, in order to increase vaccination coverage and reduce vaccination hesitancy among Black people and people from lower socioeconomic strata.

## RESUMEN

**Objetivo:**

Analizar la cobertura vacunal hasta los 24 meses de vida según color de piel en una cohorte de nacidos vivos en 2017-2018 en Natal, Rio Grande del Norte.

**Métodos:**

Encuesta poblacional realizada en 2020 y 2021. Se estimó la cobertura de vacunación hasta los 24 meses de edad, según dosis aplicadas, válidas y oportunas. La asociación bruta de raza/color se estimó calculando índice de prevalencia bruto y los respectivos intervalos de confianza del 95%, utilizando la regresión de Poisson.

**Resultados:**

De los 688 niños de cohorte seleccionada, hubo mayor cobertura entre los niños negros para las dosis aplicadas (blancos 30,5%; negros 47,8%; IC95%) y dosis válidas (blancos 25,8%; negros 40,1%; IC95%) sin significancia estadística, y menor cobertura para dosis oportunas, pauta completa sin fiebre amarilla (RP = 0,21; IC95% 0,04;0,90).

**Conclusión:**

Hubo una cobertura menos oportuna entre los niños negros en comparación con los niños blancos.

## INTRODUCTION

Immunization is considered the main intervention measure to prevent vaccine-preventable diseases, and represents the most cost-effective investment in health in the fight against infectious diseases.^1,2^ The objective of the National Immunization Program (*Programa Nacional de Imunizaç*ões - PNI), created by the Ministry of Health in 1973, is to guarantee access by everyone to immunobiological products, universally and free of charge, aiming to control and eradicate diseases that can be prevented through immunization and contribute to the reduction of child mortality.^3,4^

The state of Rio Grande do Norte has shown a reduction in vaccination coverage since 2018, not having achieved any of the targets established for vaccines as per the basic vaccination calendar schedule indicated for children up to 1 year old, such as polio. With regard to polio, 70.2% of the target population was vaccinated against it in 2016; however, in 2017 and 2019, vaccination rates were only 69.5% and 80.7%, respectively.^5^

Vaccination coverage is a health indicator used to evaluate immunization programs and access to health services, and in this regard it is essential to estimate coverage, considering administered, valid and timely doses.^6-9^ Furthermore, socioeconomic and sociodemographic factors – such as low levels of maternal education, mothers who work away from home, mothers and heads of family being of Black race/skin color – are associated with higher percentages of basic childhood vaccination schedule incompleteness.^1^ These factors are reflections of inequalities in access to health services.

It should be emphasized that race/skin color is a marker of social inequalities and is considered an important predictor of the health of a population, in addition to the various dimensions of racism being recognized as structural determinants of the morbidity and mortality profile.^10,11^ The Black population in Brazil is subject to vulnerabilities that imply difficulties in accessing health services and establish a relationship with processes of racial stigmatization.^11,12^

The National Policy on Comprehensive Health for Black People (*Política Nacional de Saúde Integral da População Negra* - PNSIPN) is an instrument aimed at guaranteeing access to health services^13^ and highlights the role of racism as a social determinant of the health of this population segment. To address the historical losses and inequalities caused by structural racism, the PNSPIN develops intersectoral programs and actions, involving multiple Ministry of Health divisions, in addition to civil initiatives and the participation of social movements.^14^

Few Brazilian studies analyze vaccination coverage according to children’s race/skin color. As such, investigating vaccination coverage according to race/skin color contributes to the planning of actions and strategies in childhood immunization services, aiming to increase vaccination coverage and reduce vaccination hesitancy, seeking to correct social inequalities related to race/skin color. The objective of this study was therefore to analyze vaccination coverage up to 24 months of age, according to the race/skin color, among children born in 2017-2018 in Natal, in the state of Rio Grande do Norte (RN), Brazil.

## METHODS

This is a population-based household survey carried out in the city of Natal/RN. The data used in the study comes from the 2020 National Vaccination Coverage Survey (*Inquérito Nacional de Cobertura Vacinal 2020* - INCV 2020) which was conducted in the urban areas of the 26 Brazilian state capitals, in the Federal District and in 12 municipalities in the interior region with more than 100,000 inhabitants, between 2020 and 2021.^15^

The study population was made up of children born alive in 2017 and 2018, registered on the Live Birth Information System (*Sistema de Informação sobre Nascidos Vivos* - SINASC), residing in the urban area of the municipality of Natal, capital of the state of Rio Grande do Norte. According to data from the 2022 Brazilian Census, Natal has 751,300 inhabitants and covers an area of 167,401 km², 99.32% of which is urbanized.^16^ In all, Natal’s municipal health network has 147 health centers providing public services, 80 of which are run by the municipal government, ten by the state government and four by the federal government; in addition, six charity health centers and 47 outsourced private health centers provide municipal services under the Brazilian National Health System (*Sistema Único de Saúde* - SUS).^17^

Sampling was probabilistic using clusters defined through three selection stages. In the first stage, the census tracts were classified into four socioeconomic strata, using data on the following socioeconomic variables: average income of heads of household; percentage of heads of household with income above 20 minimum wages; and percentage of literate heads of household, based on the 2010 Demographic Census. Once the census tracts had been identified in each socioeconomic stratum, the second stage was characterized by the formation of clusters of tracts (one or more tracts), according to the estimated number of live births from the 2017 and 2018 cohort in each census tract, so that each cluster had at least 56 cohort live births. Next, the clusters were systematically selected at random, so that eight clusters were included in each stratum. In the third stage, the sampling units were the children themselves. Using the maps of the clusters selected and the list of addresses held on the SINASC system, the interviewers went through the respective areas, looking for children from the cohorts, until the pre-established number for each stratum was reached.

The sample size was defined based on the calculations adopted for the INCV 2020. For the current study, we used 95% confidence intervals, expected vaccination coverage of 70% and a design effect of 1.4.^15^

A standardized questionnaire was used, answered by the child’s mother or guardian and filled out based on the information contained on the child’s vaccination card, which was photographed for greater data reliability. Other methodological details of the vaccination coverage survey have been described in a specific publication.^15^

The dependent variables were vaccination coverage per immunobiological product and full vaccination coverage for vaccines that should have been administered in the first year of life and for those that should have been administered after the first year of life (excluding yellow fever vaccine), as per the vaccination schedule for children aged 12 and 24 months, administered in public and/or private services. Coverage of the following immunobiological products was analyzed: Bacillus Calmette-Guérin (BCG), hepatitis B vaccine, DTcP-Hib-Hepb vaccine, poliovirus vaccine, human rotavirus vaccine, meningococcal C (MenC) vaccine, 10-valent pneumococcal vaccine (PCV10), triple viral vaccine, varicella vaccine, hepatitis A vaccine and diphtheria, tetanus and pertussis (DTP) vaccine, the doses of which were classified into administered, valid and timely doses. Yellow fever vaccine was excluded as it was not part of the immunization schedule in 2017 and 2018 in some state capital cities in the Northeast region.

 Vaccines recorded on the vaccination card or on the National Immunization Program Information System (*Sistema de Informação do Programa Nacional de Imunizações* - SI-PNI) were considered as administered doses; valid doses were doses received with a start date and adequate interval between doses; while timely doses were those received according to the national immunization schedule with a variation of ± 15 days.^7-9^

In order to calculate full vaccination coverage at 24 months, the numerator was taken to be the “number of children who completed the recommended vaccination schedule (BCG, hepatitis B vaccine, DTcP-Hib-Hepb vaccine (first, second and third dose, first booster), poliovirus vaccine (first, second and third dose, first booster), human rotavirus vaccine (first and second dose), MenC (first and second dose, first booster), PCV10 (first and second dose, first booster), triple viral (first and second dose), varicella vaccine, hepatitis A vaccine and DTP”, divided by the denominator “number of children included in the study”, multiplied by 100.

The main independent variable was the child’s race/skin color, categorized as: 1) White and 2) Black. We chose to use the “Black” race/skin color category, combining Black and mixed race children, due to the small number of Black children in the study. The “Asian” and “Indigenous” race/skin color categories were excluded from the analysis.

The independent sample characterization variables are specified below.

- Maternal characteristics: schooling in years (< 8, 9-12, 13-15, ≥ 16); age in years (< 20, 20-34, ≥ 35);
- Family characteristics: household crowding (yes – more than three people per room used for sleeping, no – up to three people per room used for sleeping); family consumption stratum (stratum A-B, stratum C-D); has used or uses private services for vaccination (yes, no); ever had difficulty in taking the child to the vaccination center (yes, no); believes that vaccines are important for children’s health (yes, no); believes that vaccines are important for neighborhood health (yes, no); and trusts vaccines distributed by the government (yes, no).

The statistical analyses were performed using STATA version 13. The sample weight was considered and the study design effect was incorporated. Vaccination coverage of administered, valid and timely doses was calculated, according to the children’s race/skin color. Crude association of race/skin color with the outcomes analyzed was estimated by calculating the crude prevalence ratio and respective 95% confidence intervals (95%CI), using Poisson regression.

The study was approved by the Research Ethics Committee of the *Instituto de Saúde Coletiva da Universidade Federal da Bahia*, as per Opinion No. 3.366.818, on June 4, 2019, and Certificate of Submission for Ethical Appraisal (*Certificado de Apresentação de Apreciação* Ética – CAAE) No. 4306919.5.0000.5030; and by the Research Ethics Committee of the *Irmandade da Santa Casa de São Paulo*, as per Opinion No. 4.380.019, on November 4, 2020, and CAAE No. 39412020.0.0000.5479. 

## RESULTS

The highest percentage of interviews that were not carried out occurred in stratum A (62.7%) and stratum B (31.6%). In stratum C (low medium) and D stratum (low), 100% of the interviews were carried out. The reason for not reaching the expected target was failure to locate the children. Eleven children were excluded from the study, as they were in the “Asian” and “Indigenous” race/skin color categories. 

Of the 904 interviews expected according to the sample size calculation, 688 were carried out, representing a sample loss of 23.9%. The characterization of the sample reveals that the majority of children of Black race/skin color were from families with a low consumption level (C-D) (80.4%) and from families that report living in a crowded household (14.0%), children of mothers with a lower level of education (24.5%), 19.9% used private vaccination services and 15.6% of mothers reported difficulty in taking their child to the vaccination center. Among Black children, 95.0% of those responsible for them reported trusting the vaccines distributed by the government, 97.0% reported the belief that vaccines are important for children’s health, and 94.5% reported the belief that vaccines are important for the health of the neighborhood (Table 1).

**Table 1:** Family and maternal characteristics (%) and respective confidence intervals (95%CI), and information about vaccination hesitancy, by race/skin color of the children taking part in the National Vaccination Coverage Survey for children born in 2017-2018, Natal, Rio Grande do Norte, Brazil, 2024 (n = 688)

Variables	Child’s race/skin color	
	**White**	**Black**
	**% (95%CI)**	**% (95%CI)**
**Child’s sex**		
Male	55.0 (43.4;66.1)	52.8 (42.7;62.8)
Female	44.9 (33.8;56.5)	47.1 (37.2;57.3)
**Maternal age group (years)**		
< 20	0.7 (0.2;2.3)	4.8 (1.1;17.4)
20-34	60.8 (48.9;71.5)	56.3 (44.9;67.0)
≥ 35	38.4 (27.7;50.3)	38.8 (27.9;51.0)
**Maternal schooling (years)**		
< 8	7.8 (4.2;14.1)	24.5 (14.6;38.2)
9-12	11.3 (5.2;22.6)	15.6 (9.3;24.9)
13-15	29.3 (18.1;43.8)	37.0 (28.0;47.1)
≥ 16	51.4 (35.0;67.4)	22.7 (11.9;88.9)
**Household crowding**		
Yes (4 or more people per bedroom)	2.5 (1.1;5.5)	14.0 (8.4;22.5)
No (1-3 people per bedroom)	97.5 (94.4;98.8)	85.9 (77.4;91.5)
**Family consumption level**		
A-B	36.1 (24.2;49.9)	19.5 (9.3;36.5)
C-D	63.8 (50.0;75.7)	80.4 (63.4;90.6)
**Use of private service for vaccination**		
Yes	36.2 (23.1;51.7)	19.9 (9.6;36.9)
**Ever had difficulty in taking the child to the vaccination center**		
Yes	29.4 (17.0;45.8)	15.6 (9.6;24.5)
**Believes that vaccines are important for children’s health**		
Yes	99.6 (98.8;99.9)	97.0 (88.2;99.2)
**Believes that vaccines are important for neighborhood health**		
Yes	99.7 (98.5;99.9)	94.5 (77.4;98.8)
**Trusts vaccines distributed by the government**		
Yes	99.7 (99.0;99.9)	95.0 (86.8;98.2)

Regarding vaccination coverage by immunobiological products that are on the national immunization schedule, it can be seen that vaccination coverage of valid doses was higher among black children in 19 doses of the immunizing agents analyzed. However, vaccination coverage of timely doses among Black children was lower than that found for White children in 20 doses of the immunizing agents analyzed. The differences found were not, however, statistically significant (Table 2).

**Table 2:** Vaccination coverage according to valid and timely doses, prevalence ratio (PR) and respective confidence intervals (95%CI), by race/skin color of the children taking part in the National Vaccination Coverage Survey for children born in 2017-2018, Natal, Rio Grande do Norte, Brazil, 2024 (n = 688)

Immunizing agent	Bivariate analysis	
	**Valid doses**	**Timely doses**
	**PR (95%CI)**	**PR (95%CI)**
Bacillus Calmette-Guérin	1.09 (0.83;1.44)	0.84 (0.51;1.40)
Hepatitis B vaccine	1.09 (0.83;1.45)	0.84 (0.51;1.38)
First dose of DTcP-Hib-Hepb vaccine	1.04 (0.91;1.19)	0.82 (0.49;1.38)
Second dose of DTcP-Hib-Hepb vaccine	1.01 (0.88;1.17)	0.93 (0.55;1.58)
Third dose of DTcP-Hib-Hepb vaccine	1.09 (0.91;1.29)	0.76 (0.47;1.25)
First dose of poliovirus vaccine	1.04 (0.91;1.19)	0.83 (0.50;1.38)
Second dose of poliovirus vaccine	1.02 (0.89;1.17)	0.92 (0.55;1.54)
Third dose of poliovirus vaccine	1.17 (0.95;1.44)	0.64 (0.36;1.12)
First poliovirus vaccine booster	1.15 (0.89;1.50)	0.69 (0.37;1.28)
First dose of human rotavirus vaccine	1.02 (0.88;1.17)	0.94 (0.59;1.50)
Second dose of human rotavirus vaccine	1.10 (0.86;1.40)	0.81 (0.55;1.21)
First dose of meningococcal C vaccine	1.03 (0.90;1.19)	0.82 (0.48;1.42)
Second dose of meningococcal C vaccine	1.01 (0.88;1.17)	0.92 (0.55;1.53)
First meningococcal C vaccine booster	1.03 (0.86;1.24)	0.92 (0.62;1.37)
First dose of pneumococcal vaccine	1.03 (0.89;1.18)	0.88 (0.52;1.49)
Second dose of pneumococcal vaccine	1.02 (0.88;1.17)	0.86 (0.50;1.47)
First pneumococcal vaccine booster	1.09 (0.83;1.43)	0.81 (0.44;1.49)
First dose of triple viral vaccine	0.98 (0.83;1.17)	1.02 (0.53;1.97)
Second dose of triple viral vaccine	0.99 (0.82;1.19)	0.99 (0.61;1.60)
Varicella vaccine	1.02 (0.89;1.17)	0.88 (0.56;1.37)
Hepatitis A vaccine	0.95 (0.79;1.12)	1.14 (0.63;2.05)
First diphtheria, tetanus and pertussis vaccine booster	1.06 (0.63;1.34)	0.84 (0.46;1.51)

The analysis of full vaccination coverage shows that Black children have greater coverage both with regard to administered doses (White children, 30.5%, versus Black children, 47.8%) and with regard to valid doses (White children, 25.8% versus Black children, 40.1%), although there was no statistically significant difference (Table 3). 

**Table 3:** Full vaccination coverage (%) up to 24 months old, with prevalence ratio and respective confidence intervals (95%CI), according to dose classification as administered, valid and timely, by race/skin color of the children taking part in the National Vaccination Coverage Survey for children born in 2017-2018, Natal, Rio Grande do Norte, Brazil, 2024 (n = 688)

Dose classification	Race/skin color	Coverage of vaccines recommended in the first year of life		Coverage of vaccines that should be taken after the first year of life		Full coverage	
		**% (95%CI)**	**Crude PR (95%CI)**	**% (95%CI)**	**Crude PR (95%CI)**	**% (95%CI)**	**Crude PR (95%CI)**
Administered doses	White	45.9% (33.0;59.5)	1.30 (0.87;1.95)	46.5% (34.7;58.6)	1.23 (0.84;1.79)	30.5% (18.4;46.0)	1.56 (0.89;2.76)
	Black	60.1% (48.1;71.0)		57.2% (44.5;68.9)		47.8% (36.4;59.5)	
Valid doses	White	41.1% (29.9;53.3)	1.29 (0.85;1.95)	34.8% (21.6;50.8)	1.48 (0.86;2.52)	25.8% (15.8;39.2)	1.55 (0.85;2.81)
	Black	53.2% (40.9;65.1)		51.6% (39.1;64.0)		40.1% (28.6;52.8)	
Timely doses	White	14.5% (8.1;24.6)	1.16 (0.54;2.46)	3.4% (1.7;6.8)	0.51 (0.15;1.67)	7.2% (2.6;18.5)	0.21 (0.04;0.90)
	Black	16.8% (10.5;25.7)		1.7% (0.7;4.2)		1.5% (0.5;4.3)	

However, with regard to timely doses, there was lower vaccination coverage among Black children for vaccines that should be taken after the first year of life and for full coverage up to 24 months old. There was a significant difference between Black children and White children for full coverage up to 24 months old (PR = 0.21; 95%CI 0.04;0.90), considering the timely doses (Table 3).

## DISCUSSION

The study found lower coverage, considering timely doses for multiple-dose vaccines and the full basic vaccination schedule (not including yellow fever vaccine), among Black children, in the cohort born between 2017 and 2018 in the city of Natal, showing that racial inequalities represented barriers to the completion of the vaccination schedule by 24 months old.

The findings of this study show a vaccination profile in Brazil in which population groups from lower socioeconomic strata have poorer vaccination opportunities. Prevalence of timely full vaccination coverage was 79% lower among Black children when compared to White children.

In contrast, a study carried out in the city of Pelotas/RS, in 2015, showed that the group from higher socioeconomic strata had higher risk of incomplete vaccination;^18^ in the population-based survey, carried out in Brazilian state capitals in 2012, children’s race/skin color was not associated with incomplete vaccination.^19^

Corroborating the findings of the present study, a household survey carried out in São Luís/MA, in 2006, found that, even after adjustment for socioeconomic factors, differences in coverage according to race/skin color remained, highlighting that Black children face greater difficulties in getting vaccinated.^20^

This unfair and avoidable inequality can be explained by structural racism that manifests itself in the healthcare field. This phenomenon can materialize through internalized prejudices, stereotypes and negative feelings linked to the racial or ethnic characteristics of a given group; through interpersonal manifestation of discriminatory behaviors and practices, which exclude and diminish these groups, attributing less value to them or considering them to have no value in relation to other groups; and, as a structural component, legitimized and practiced by organizations, policies and standards, through unfair, discriminatory, negligent treatment, with disadvantages in access to benefits and delays in implementing actions and policies that would favor their victims.^21^

In the present study, most Black children belonged to classes C and D, had mothers with low education levels, and showed greater vaccination hesitancy. However, there was no significant difference when compared to the White children analyzed in this study. 

In London, United Kingdom, a study assessed the overall effect of maternal education on childhood vaccination and indicated that there is a direct relationship between maternal education and childhood vaccination, and that there was a significant difference in the vaccination coverage of children born to literate and non-literate mothers, showing that higher levels of education had a positive impact on adherence to vaccination.^22^ Women with higher levels of education tend to seek health services more and be more aware of prevention through immunization, compared to women with no schooling.^23^

Among the families of Black children in this study, there was a higher percentage of vaccination hesitancy, although not statistically different when compared to White children. The phenomenon of vaccination hesitancy may be associated with disbelief in vaccines, vaccine shortages in health centers, lack of knowledge related to vaccines and vaccine-preventable diseases, and difficulty in accessing health services.^4,24^

Still on this topic, a study^2^ reports that the false perception that it is no longer necessary to vaccinate one’s children, believing that diseases disappear over time, can lead parents to hesitate with regard to vaccination, and that these factors are associated with the determinants of vaccination coverage.

Furthermore, the spread of false news about adverse childhood vaccine events is also present in Black populations and those with lower education levels, making it even more difficult to achieve a full vaccination schedule.^1^

In our study, we found that, among Black children, there was a higher proportion of household crowding, which indicates that this is a marker of poorer socioeconomic conditions. Another study^25^ found that children from the poorest households were 36% more likely to not be immunized than children from the wealthiest households. According to another survey,^19^ due to household crowding, there is a probability that the person responsible for the child will have difficulty in going to the health center, as they do not have time available to get their child vaccinated, which may compromise the child’s vaccination schedule.

Another important finding of the present study was low vaccination coverage with timely doses among Black children. It is important to highlight that, when the majority of children are vaccinated in a timely manner, unvaccinated individuals and those who have failed vaccinations are also protected. In this context, an indicator that deserves to be highlighted is vaccination delay, which is defined as not receiving the dose in a timely manner, as its results can affect the vaccinated population, leaving them unprotected and interfering with herd immunity.^26,^ ^27^

The explanation for this fact is that, traditionally, the most advantaged portion of the population receives more and better health interventions, resulting in greater vaccination coverage in this group. Vaccination coverage has been decreasing over the years in high-income countries, which indicates that factors that favor access and use of services may be converging on the phenomenon of vaccination hesitancy. However, there are signs of a reversal of this pattern in some low- and middle-income countries, where coverage is increasing among the poorest and decreasing among the wealthiest.^8,19,28^

This specific finding of this study shows that inequality in vaccination coverage follows the expected pattern of greater coverage among those with better socioeconomic conditions. However, there are already signs of a reversal of this pattern, as no significant differences were found in vaccination coverage between Black and White children for the majority of forms of coverage analyzed.

Identifying these disadvantages according to race/skin color also fills a knowledge gap in Brazil. Racial inequalities in health indicators have contributed considerably to the increase in social disparities.^1,11,20,25^

The World Health Organization (WHO) understands the importance of research related to vaccination, aiming to reduce child mortality, and highlights the relevance of high-quality data for monitoring, prevention and creation of strategies aimed at better distribution and administration of vaccines, in addition to support for children’s families.^29^

As provided for by article 14 of the Child and Adolescent Statute, “vaccination of children is mandatory in cases recommended by health authorities”. Furthermore, it is the duty of the family, society and the State to ensure, as a priority, the implementation of rights relating to the life and health of children and adolescents.^30^

In general, standing out among the strengths of this research, which is a population-based study, are the sample size and the sampling process, as well as the strategy adopted to collect information on vaccination status, such as direct extraction from vaccination records and photographs of the children’s vaccination cards, which can serve to reduce possible measurement biases.

The limitations of this study are related to possible information bias, as there was the possibility that the survey respondent was not the parent or guardian of the child; and participation bias, considering that there was a higher percentage of losses due to not finding the address, due to households being closed and due to refusal among families in economic classes A (60.4%) and B (47.5%). This may have affected the calculation of vaccination coverage according to race/skin color, as a higher concentration of children of White race/skin color was found in these strata. Another limitation concerns grouping together race/skin color categories (Black and mixed race) into a single category and the exclusion of individuals in the Asian and Indigenous race/skin color categories. Given the low number of participants in these categories in the sample, creating single categories could cause greater statistical variability and lead to wider and less precise confidence intervals when estimating population parameters. 

Our results reflect the need to improve the Brazilian health system, with a view to correcting the inequities highlighted. Among promotion and prevention actions, we highlight that educational actions must address information about the importance of childhood vaccination; and communication must be continuous, in simple, consistent and culturally appropriate language.

In addition to the participation of family members, the child health care network must guarantee access to immunobiological products, as well as equipment to preserve their quality; the training of its professionals regarding the appropriate administration of vaccines and post-vaccination care guidelines and compliance with the vaccination plan; in addition to offering opportunities for vaccine updates for susceptible people, thus promoting collective protection against vaccine-preventable diseases.

Strengthening the strategic actions of the PNI could increase access to health services, as well as the supply and use of these services for that part of the population that needs it most, with a view to reducing racial/social inequalities. Furthermore, there is a need to carry out assessments of the quality of care offered and the performance of the health system, based on issues such as adequacy, continuity, acceptability, effectiveness, efficiency, safety and respect for individuals’ rights.
