# Supplementary material for: Vaccination coverage according to race or skin color in children born in 2017-2018 in Natal, Rio Grande do Norte, Brazil: a population survey
Source: Epidemiol Serv Saude. 2024 Dec 13;33(spe2):e20231310. doi: 10.1590/S2237-96222024v33e20231310.especial2.en (PMC11654711; doi:10.1590/S2237-96222024v33e20231310.especial2.en)
Supplement: Translation

# Translation: Cobertura vacinal de acordo com o quesito raça/cor da pele em crianças nascidas em 2017-2018 em Natal, Rio Grande do Norte: inquérito populacional

**DOI:** 10.1590/S2237-96222024v33e20231310.especial2.pt

**Authors:** Eliene Roberta Alves dos Santos, Isabelle Ribeiro Barbosa, José Cássio de Moraes, Ana Paula França, Carla Magda Allan Santos Domingues, Maria da Gloria Teixeira, Héllyda de Souza Bezerra, Nayre Beatriz Martiniano de Medeiros, Mayonara Fabíola Silva Araújo, Fábia Cheyenne Gomes de Morais Fernandes, Arthur Alexandrino, Ricardo Andrade Bezerra, Grupo ICV 2020, Adriana Ilha da Silva, Alberto Novaes Ramos, Ana Paula França, Andrea de Nazaré Marvão Oliveira, Antonio Fernando Boing, Carla Magda Allan Santos Domingues, Consuelo Silva de Oliveira, Ethel Leonor Noia Maciel, Ione Aquemi Guibu, Isabelle Ribeiro Barbosa, Jaqueline Caracas Barbosa, Jaqueline Costa Lima, José Cássio de Moraes, Karin Regina Luhm, Karlla Antonieta Amorim Caetano, Luisa Helena de Oliveira Lima, Maria Bernadete de Cerqueira Antunes, Maria da Gloria Teixeira, Maria Denise de Castro Teixeira, Maria Fernanda de Sousa Oliveira Borges, Rejane Christine de Sousa Queiroz, Ricardo Queiroz Gurgel, Rita Barradas Barata, Roberta Nogueira Calandrini de Azevedo, Sandra Maria do Valle Leone de Oliveira, Sheila Araújo Teles, Silvana Granado Nogueira da Gama, Sotero Serrate Mengue, Taynãna César Simões, Valdir Nascimento, Wildo Navegantes de Araújo

**Affiliations:** 1 Universidade Federal do Rio Grande do Norte, Programa de Pós-Graduação em Saúde Coletiva, Santa Cruz, RN, Brasil; 2 Santa Casa de São Paulo, Faculdade de Ciências Médicas, São Paulo, SP, Brasil; 3 Organização Pan-Americana da Saúde, Brasília, DF, Brasil; 4 Universidade Federal da Bahia, Instituto de Saúde Coletiva, Salvador, BA, Brasil; 5 Universidade Federal do Rio Grande do Norte, Programa de Pós-Graduação em Saúde Coletiva, Natal, RN, Brasil; Universidade Federal do Espírito Santo, Vitória, ES, Brasil; Universidade Federal do Ceará, Departamento de Saúde Comunitária, Fortaleza, CE, Brasil; Faculdade Ciências Médicas Santa Casa de São Paulo, São Paulo, SP, Brasil; Secretaria de Estado da Saúde do Amapá, Macapá, AP, Brasil; Universidade Federal de Santa Catarina, SC, Brasil; Organização Pan-Americana da Saúde, Brasília, DF, Brasil; Instituto Evandro Chagas, Belém, PA, Brasil; Faculdade de Ciências Médicas Santa Casa de São Paulo, Departamento de Saúde Coletiva, São Paulo, SP, Brasil; Universidade Federal de Mato Grosso, Cuiabá, MT, Brasil; Universidade Federal do Paraná, Curitiba, PR, Brasil; Universidade Federal de Goiás, Goiânia, GO, Brasil; Universidade Federal do Piauí, Teresina, PI, Brasil; Universidade de Pernambuco, Faculdade de Ciências Médicas, Recife, PE, Brasil; Instituto de Saúde Coletiva, Universidade Federal da Bahia, Salvador, BA, Brasil; Secretaria de Estado da Saúde de Alagoas, Maceió, AL, Brasil; Universidade Federal do Acre, Rio Branco, AC, Brasil; Universidade Federal do Maranhão, Departamento de Saúde Pública, São Luís, MA, Brasil; Universidade Federal de Sergipe, Aracaju, SE, Brasil; Secretaria Municipal de Saúde, Boa Vista, RR, Brasil; Fundação Oswaldo Cruz, Mato Grosso do Sul, Campo Grande, MS, Brasil; Fundação Oswaldo Cruz, Escola Nacional de Saúde Pública Sergio Arouca, Rio de Janeiro, RJ, Brasil; Universidade Federal do Rio Grande do Sul, Porto Alegre, RS, Brasil; Fundação Oswaldo Cruz, Instituto de Pesquisa René Rachou, Belo Horizonte, MG, Brasil; Secretaria de Desenvolvimento Ambiental de Rondônia, Porto Velho, RO, Brasil; Universidade de Brasília, Brasília, DF, Brasil

**Keywords:** Cobertura Vacinal, Disparidades Socioeconômicas em Saúde, Acesso aos Serviços de Saúde, Saúde das Minorias Étnicas, Determinantes Sociais da Saúde, Inquéritos Epidemiológicos

## Abstract

**Objetivo:**

Analisar a cobertura vacinal até os 24 meses de vida, segundo raça/cor da pele, na coorte de nascidos vivos em 2017-2018 em Natal, Rio Grande do Norte.

**Métodos:**

Inquérito de base populacional realizado em 2020 e 2021. Estimaram-se as coberturas vacinais até os 24 meses de idade, segundo doses aplicadas, válidas e oportunas. A associação bruta da raça/cor da pele foi estimada pelo cálculo da razão de prevalência bruta e respectivos intervalos de confiança de 95% (IC_95_%), utilizando-se regressão de Poisson.

**Resultados:**

Das 688 crianças da coorte selecionadas, houve maior cobertura entre as crianças negras para as doses aplicadas (brancas 30,5%; negras 47,8%; IC_95_%) e válidas (brancas 25,8%; negras 40,1%; IC_95_%), sem significância estatística, e menor cobertura para as doses oportunas, no esquema completo sem febre amarela (RP = 0,21; IC_95_% 0,04;0,90).

**Conclusão:**

Evidenciou-se menor cobertura oportuna entre crianças negras, comparadas com crianças brancas.

## Contribuições do estudo

**Principais resultados:**

Constatou-se que as desigualdades raciais e as disparidades sociais refletiram na cobertura vacinal das crianças de pele negra do estado do Rio Grande do Norte, evidenciando menor prevalência do esquema vacinal oportuno e completo entre essas crianças.

**Implicações para os serviços:**

Os resultados demonstram a necessidade de reforçar as políticas públicas equitativas e a realização de práticas que busquem melhorar as coberturas vacinais, diminuindo assim as desigualdades raciais no âmbito da imunização em crianças.

**Perspectivas:**

Gestores poderão realizar o planejamento de ações e estratégias nos serviços de imunização infantil, para o aumento da cobertura vacinal e a redução da hesitação em se vacinar entre pessoas negras e de condições socioeconômicas inferiores.

## INTRODUÇÃO

A imunização é considerada a principal medida de intervenção para prevenir doenças imunopreveníveis, e representa o investimento em saúde com melhor custo-benefício no combate às doenças infecciosas.^1,2^ O Programa Nacional de Imunizações (PNI), criado pelo Ministério da Saúde, em 1973, tem por objetivo garantir o acesso de todos aos imunobiológicos, de forma universal e gratuita, visando controlar e erradicar doenças preveníveis pela imunização e contribuir para a redução da mortalidade infantil.^3,4^

O estado do Rio Grande do Norte tem apresentado redução nas coberturas vacinais desde o ano de 2018, não tendo atingido nenhuma das metas estabelecidas para as vacinas do calendário vacinal básico indicadas para as crianças de até 1 ano de idade, a exemplo da poliomielite. Contra essa doença, 70,2% da população-alvo foi vacinada em 2016; porém, em 2017 e 2019, as taxas de vacinação foram de apenas 69,5% e 80,7%, respectivamente.^5^

A cobertura vacinal é um indicador de saúde utilizado para avaliar os programas de imunização e o acesso aos serviços de saúde, e nesse quesito é essencial estimar as coberturas, considerando-se as doses aplicadas, válidas e oportunas.^6-9^ Além disso, fatores socioeconômicos e sociodemográficos – como baixa escolaridade materna, mães que trabalham fora dos seus lares, a cor da pele negra da mãe e do chefe de família – estão associados a maiores percentuais de incompletude do calendário vacinal básico infantil.^1^ Esses fatores são reflexos das desigualdades de acesso a serviços de saúde.

Ressalta-se que a raça/cor da pele é um marcador de desigualdades sociais e é considerada um importante preditor da saúde de uma população, além das diversas dimensões do racismo serem reconhecidas como determinantes estruturais do perfil de morbimortalidade.^10,11^ A população negra no Brasil está submetida a vulnerabilidades que implicam dificuldades de acesso aos serviços de saúde e estabelecem relação com processos de estigmatização racial.^11,12^

A Política Nacional de Saúde Integral da População Negra (PNSIPN) é um instrumento voltado a garantir o acesso aos serviços de saúde^13^ e destaca o papel do racismo como determinante social para a saúde desse segmento populacional. Para enfrentar os prejuízos históricos e as desigualdades causadas pelo racismo estrutural, a PNSPIN inclui programas e ações intersetoriais, envolvendo múltiplos órgãos do Ministério da Saúde, além de iniciativas civis e da participação de movimentos sociais.^14^

São escassos os estudos brasileiros que analisam a cobertura vacinal segundo o quesito raça/cor da pele de crianças. Dessa forma, a investigação da cobertura vacinal segundo raça/cor da pele contribui para o planejamento de ações e estratégias nos serviços de imunização infantil, visando ao aumento da cobertura vacinal e à redução da hesitação vacinal, buscando-se corrigir as desigualdades sociais relacionadas à raça/cor da pele. Assim, o objetivo deste estudo foi analisar a cobertura vacinal até os 24 meses de vida, de acordo com o quesito raça/cor da pele, em crianças nascidas em 2017-2018 em Natal, no estado do Rio Grande do Norte.

## MÉTODOS

Trata-se de um inquérito domiciliar de base populacional realizado no município de Natal/RN. Os dados utilizados no estudo são provenientes do Inquérito Nacional de Cobertura Vacinal 2020 (INCV 2020) realizado nas áreas urbanas das 26 capitais brasileiras, no Distrito Federal e em 12 municípios do interior acima de 100 mil habitantes, entre 2020 e 2021.^15^

A população de estudo foi composta por nascidos vivos em 2017 e 2018, registrados no Sistema de Informação sobre Nascidos Vivos (Sinasc), residentes na área urbana do município de Natal, capital do estado do Rio Grande do Norte. Segundo dado do Censo brasileiro de 2022, Natal possui 751.300 habitantes e território de 167,401 km², com 99,32% de sua área urbanizada.^16^ A rede municipal de saúde de Natal conta com 147 unidades de saúde, sendo 80 públicas municipais, dez estaduais e quatro federais; além disso, seis unidades filantrópicas e 47 unidades privadas contratadas prestam serviço municipal ao Sistema Único de Saúde (SUS).^17^

A amostragem foi probabilística por conglomerados em três estágios de seleção. No primeiro estágio, os setores censitários foram classificados em quatro estratos socioeconômicos, utilizando-se dados das variáveis socioeconômicas renda média dos responsáveis pelas famílias, porcentagem de responsáveis com renda superior a 20 salários mínimos e porcentagem de responsáveis alfabetizados, do Censo Demográfico de 2010. Uma vez identificados os setores censitários em cada estrato socioeconômico, o segundo estágio caracterizou-se pela formação de conglomerados de setores (um ou mais setores), de acordo com o número estimado de nascidos vivos da coorte de 2017 e 2018 em cada setor censitário, de modo que cada conglomerado tivesse pelo menos 56 nascidos vivos. Em seguida, procedeu-se ao sorteio sistemático dos conglomerados, de modo a serem incluídos oito deles em cada estrato. No terceiro estágio, a unidade de amostragem foi a criança. Com os mapas dos conglomerados sorteados e a listagem dos endereços, obtidos no Sinasc, os entrevistadores percorreram as áreas respectivas, buscando as crianças das coortes, até se atingir o número preestabelecido para cada estrato.

O tamanho da amostra foi definido a partir dos cálculos adotados no INCV 2020. Para o estudo atual, utilizaram-se os parâmetros de 95% de confiança, uma cobertura vacinal esperada de 70% e efeito de desenho de 1,4.^15^

Utilizou-se questionário padronizado respondido pela mãe ou responsável pela criança e preenchido tomando-se por base as informações contidas na caderneta de vacinação, que foi fotografada para maior fidedignidade dos dados. Outros detalhes metodológicos do inquérito de cobertura vacinal foram descritos em publicação específica.^15^

As variáveis dependentes foram as coberturas vacinais por imunobiológico e as coberturas vacinais completas para as vacinas que deveriam ter sido administradas no primeiro ano de vida e para as que deveriam ter sido administradas depois do primeiro ano de vida (sem a vacina contra febre amarela), do calendário vacinal de crianças aos 12 e 24 meses de vida, administrados nos serviços públicos e/ou privados. Foram analisadas as coberturas dos seguintes imunobiológicos: bacilo de Calmette-Guérin (BCG), vacina contra hepatite B, vacina pentavalente, vacinas contra poliomielite, vacina contra rotavírus humano, vacina meningocócica C (MenC), vacina pneumocócica 10-valente (VPC10), tríplice viral, vacina contra varicela, vacina contra hepatite A e vacina contra difteria, tétano e pertússis (DTP), cujas doses foram classificadas em doses aplicadas, válidas e oportunas. A vacina contra febre amarela foi excluída, por não fazer parte do calendário de imunização em 2017 e 2018 em algumas capitais da região Nordeste.

Foram considerados como doses aplicadas aquelas vacinas anotadas na caderneta de vacinação ou no Sistema de Informação do Programa Nacional de Imunizações (SI-PNI); doses válidas foram as doses recebidas com data de início e intervalo adequado entre as doses; e doses oportunas, as doses recebidas de acordo com o calendário nacional de imunização com variação de ± 15 dias.^7-9^

Para o cálculo da cobertura vacinal completa aos 24 meses, considerou-se como numerador o “número de crianças que completaram o esquema vacinal preconizado (BCG, vacina contra hepatite B, vacina pentavalente [primeira, segunda e terceira dose, primeiro reforço]), vacinas contra poliomielite (primeira, segunda e terceira dose, primeiro reforço), vacina contra rotavírus humano (primeira e segunda dose), MenC (primeira e segunda dose, primeiro reforço), VPC10 (primeira e segunda dose, primeiro reforço), tríplice viral (primeira e segunda dose), vacina contra varicela, vacina contra hepatite A e DTP”, dividido pelo denominador “número de crianças incluídas no estudo”, multiplicado por 100.

A variável independente principal foi a raça/cor da pele da criança, categorizada em: 1) branca e 2) negra (pretas e pardas). Optou-se por utilizar a categoria “negra”, com a junção de crianças pretas e pardas, devido ao pequeno número de crianças de cor preta no estudo. As categorias “amarela” e “indígena” foram excluídas da análise.

As variáveis independentes de caracterização da amostra são especificadas a seguir.

- Características maternas: escolaridade em anos (< 8, 9-12, 13-15, ≥ 16); idade em anos (< 20, 20-34, ≥ 35);

- Características da família: aglomeração familiar (sim – mais de três pessoas residentes por cômodo utilizado para dormitório, não – até três pessoas residentes por cômodo utilizado para dormitório); estrato de consumo familiar (estrato A-B, estrato C-D); fez ou faz uso de serviço privado para vacinação (sim, não); já teve dificuldade de levar a criança ao posto de vacinação (sim, não); acredita que as vacinas são importantes para a saúde da criança (sim, não); acredita que as vacinas são importantes para a saúde do bairro (sim, não); e confia nas vacinas distribuídas pelo governo (sim, não).

As análises estatísticas foram realizadas utilizando-se o STATA, versão 13. Foi considerado o peso amostral e incorporado o efeito do desenho do estudo. Procedeu-se ao cálculo das coberturas vacinais de doses aplicadas, válidas e oportunas, de acordo com a raça/cor da pele da criança. A associação bruta da raça/cor da pele com os desfechos analisados foi estimada pelo cálculo da razão de prevalência bruta e respectivos intervalos de confiança de 95% (IC_95_%), utilizando-se regressão de Poisson.

A pesquisa foi aprovada pelos Comitês de Ética em Pesquisa com Seres Humanos do Instituto de Saúde Coletiva da Universidade Federal da Bahia, sob parecer n^o^ 3.366.818, em 4 de junho de 2019, com Certificado de Apresentação de Apreciação Ética (CAAE) 4306919.5.0000.5030; e da Irmandade da Santa Casa de São Paulo, sob parecer n^o^ 4.380.019, em 4 de novembro de 2020, com CAAE 39412020.0.0000.5479. 

## RESULTADOS

O maior percentual de entrevistas não efetuadas ocorreu nos estratos A (62,7%) e B (31,6%). Nos estratos C (médio baixo) e D (baixo), o percentual de entrevistas realizadas foi de 100%. O motivo para não atingirmos a meta prevista foi a não localização das crianças. Foram excluídas 11 crianças do estudo, que pertenciam às categorias “amarela” e “indígena”. 

Das 904 entrevistas previstas pela amostragem, 688 foram realizadas, o que representou a perda amostral de 23,9%. A caracterização da amostra indica que as crianças de raça/cor da pele negra (pretas e pardas) são em sua maioria de família de baixo nível de consumo (C-D) (80,4%) e de famílias que relatam viver em aglomeração domiciliar (14,0%), filhos de mães de menor nível de escolaridade (24,5%); 19,9% fizeram uso de serviços privados de vacinação e 15,6% das mães relataram dificuldade em levar a criança ao posto de vacinação. Entre as crianças negras, 95,0% dos responsáveis relataram confiança nas vacinas distribuídas pelo governo; 97,0% relataram a crença de que as vacinas são importantes para a saúde da criança; e 94,5%, a crença de que as vacinas são importantes para a saúde do bairro (Tabela 1).

**Tabela 1:** Características da família, maternas e informação sobre a hesitação vacinal, em (%) e com intervalos de confiança de 95% (IC95%), de acordo com raça/cor da pele das crianças participantes do Inquérito Nacional de Cobertura vacinal de crianças nascidas em 2017-2018, Natal, Rio Grande do Norte, 2024 (n = 688)

Variáveis	Raça/cor da pele da criança	
	**Branca**	**Negra**
	**% (IC**_95_ **%)**	**% (IC**_95_ **% )**
**Sexo da criança**		
Masculino	55,0 (43,4;66,1)	52,8 (42,7;62,8)
Feminino	44,9 (33,8;56,5)	47,1 (37,2;57,3)
**Faixa etária da mãe (anos)**		
< 20	0,7 (0,2;2,3)	4,8 (1,1;17,4)
20-34	60,8 (48,9;71,5)	56,3 (44,9;67,0)
≥ 35	38,4 (27,7;50,3)	38,8 (27,9;51,0)
**Escolaridade da mãe (anos)**		
< 8	7,8 (4,2;14,1)	24,5 (14,6;38,2)
9-12	11,3 (5,2;22,6)	15,6 (9,3;24,9)
13-15	29,3 (18,1;43,8)	37,0 (28,0;47,1)
≥ 16	51,4 (35,0;67,4)	22,7 (11,9;88,9)
**Aglomeração familiar**		
Sim (4 ou mais pessoas/cômodo)	2,5 (1,1;5,5)	14,0 (8,4;22,5)
Não (1 a 3 pessoas/cômodo)	97,5 (94,4;98,8)	85,9 (77,4;91,5)
**Nível de consumo familiar**		
A-B	36,1 (24,2;49,9)	19,5 (9,3;36,5)
C-D	63,8 (50,0;75,7)	80,4 (63,4;90,6)
**Uso de serviço privado para vacinação**		
Sim	36,2 (23,1;51,7)	19,9 (9,6;36,9)
**Já teve dificuldade de levar a criança ao posto de vacinação**		
Sim	29,4 (17,0;45,8)	15,6 (9,6;24,5)
**Acredita que as vacinas são importantes para a saúde da criança**		
Sim	99,6 (98,8;99,9)	97,0 (88,2;99,2)
**Acredita que as vacinas são importantes para a saúde do bairro**		
Sim	99,7 (98,5;99,9)	94,5 (77,4;98,8)
**Confia nas vacinas distribuídas pelo governo**		
Sim	99,7 (99,0;99,9)	95,0 (86,8;98,2)

Em relação à cobertura vacinal por imunobiológico incluído no calendário nacional de imunização, observa-se que as coberturas vacinais de doses válidas foram maiores entre as crianças de cor negra em 19 doses dos imunizantes analisados. Todavia, a cobertura vacinal de doses oportunas em crianças negras foi inferior às observadas para as crianças brancas em 20 doses dos imunizantes analisados. As diferenças observadas, contudo, não foram estatisticamente significativas (Tabela 2).

**Tabela 2:** Cobertura vacinal com razão de prevalência (RP) e intervalos de confiança de 95% (IC95%), segundo doses válidas e oportunas, de acordo com raça/cor da pele das crianças participantes do Inquérito Nacional de Cobertura vacinal de crianças nascidas em 2017-2018, Natal, Rio Grande do Norte, 2024 (n = 688)

Imunizante	Análise bivariada	
	**Doses válidas**	**Doses oportunas**
	**RP (IC**_95_ **%** **)**	**RP (IC**_95_ **%** **)**
Bacilo Calmette-Guérin	1,09 (0,83;1,44)	0,84 (0,51;1,40)
Vacina contra hepatite B	1,09 (0,83;1,45)	0,84 (0,51;1,38)
Primeira dose da vacina pentavalente	1,04 (0,91;1,19)	0,82 (0,49;1,38)
Segunda dose da vacina pentavalente	1,01 (0,88;1,17)	0,93 (0,55;1,58)
Terceira dose da vacina pentavalente	1,09 (0,91;1,29)	0,76 (0,47;1,25)
Primeira dose da vacina contra poliomielite	1,04 (0,91;1,19)	0,83 (0,50;1,38)
Segunda dose da vacina contra poliomielite	1,02 (0,89;1,17)	0,92 (0,55;1,54)
Terceira dose da vacina contra poliomielite	1,17 (0,95;1,44)	0,64 (0,36;1,12)
Primeiro reforço da vacina contra poliomielite	1,15 (0,89;1,50)	0,69 (0,37;1,28)
Primeira dose da vacina contra rotavírus humano	1,02 (0,88;1,17)	0,94 (0,59;1,50)
Segunda dose da vacina contra rotavírus humano	1,10 (0,86;1,40)	0,81 (0,55;1,21)
Primeira dose da vacina meningocócica C	1,03 (0,90;1,19)	0,82 (0,48;1,42)
Segunda dose da vacina meningocócica C	1,01 (0,88;1,17)	0,92 (0,55;1,53)
Primeiro reforço da vacina meningocócica C	1,03 (0,86;1,24)	0,92 (0,62;1,37)
Primeira dose da vacina pneumocócica	1,03 (0,89;1,18)	0,88 (0,52;1,49)
Segunda dose da vacina pneumocócica	1,02 (0,88;1,17)	0,86 (0,50;1,47)
Primeiro reforço da vacina pneumocócica	1,09 (0,83;1,43)	0,81 (0,44;1,49)
Primeira dose da tríplice viral	0,98 (0,83;1,17)	1,02 (0,53;1,97)
Segunda dose da tríplice viral	0,99 (0,82;1,19)	0,99 (0,61;1,60)
Vacina contra varicela	1,02 (0,89;1,17)	0,88 (0,56;1,37)
Vacina contra hepatite A	0,95 (0,79;1,12)	1,14 (0,63;2,05)
Primeiro retorço da vacina contra difteria, tétano e pertússis	1,06 (0,63;1,34)	0,84 (0,46;1,51)

A análise da cobertura vacinal completa mostra que as crianças negras possuem maior cobertura tanto nas doses aplicadas (brancas, 30,5%, *versus* negras, 47,8%) como nas doses válidas (brancas, 25,8% *versus* negras, 40,1%), porém não houve diferença estatisticamente significativa (Tabela 3). 

**Tabela 3:** Cobertura (%) vacinal completa até os 24 meses de vida, com com razão de prevalência (RP) e intervalos de confiança de 95% (IC95%), segundo classificação da dose em aplicada, válida e oportuna, de acordo com a raça/cor da pele das crianças participantes do Inquérito Nacional de Cobertura Vacinal de crianças nascidas em 2017-2018, Natal, Rio Grande do Norte, 2024 (n = 688)

Classificação das doses	Raça/cor da pele	Coberturadas vacinas recomendadas no primeiro ano de vida		Cobertura das vacinas que deveriam ser tomadas depois do primeiro ano de vida		Cobertura completa	
		**% (IC**_95_ **%** **)**	**RP bruta (IC**_95_ **%** **)**	**% (IC**_95_ **%** **)**	**RP bruta (IC**_95_ **%** **)**	**% (IC**_95_ **%** **)**	**RP bruta (IC**_95_ **%** **)**
Doses aplicadas	Branca	45,9% (33,0;59,5)	1,30 (0,87;1,95)	46,5% (34,7;58,6)	1,23 (0,84;1,79)	30,5% (18,4;46,0)	1,56 (0,89;2,76)
	Negra	60,1% (48,1;71,0)		57,2% (44,5;68,9)		47,8% (36,4;59,5)	
Doses válidas	Branca	41,1% (29,9;53,3)	1,29 (0,85;1,95)	34,8% (21,6;50,8)	1,48 (0,86;2,52)	25,8% (15,8;39,2)	1,55 (0,85;2,81)
	Negra	53,2% (40,9;65,1)		51,6% (39,1;64,0)		40,1% (28,6;52,8)	
Doses oportunas	Branca	14,5% (8,1;24,6)	1,16 (0,54;2,46)	3,4% (1,7;6,8)	0,51 (0,15;1,67)	7,2% (2,6;18,5)	0,21 (0,04;0,90)
	Negra	16,8% (10,5;25,7)		1,7% (0,7;4,2)		1,5% (0,5;4,3)	

No entanto, em relação às doses oportunas, houve menor cobertura vacinal entre as crianças negras para as vacinas que deveriam ser tomadas depois do primeiro ano de vida e para a cobertura completa até os 24 meses de vida. Houve diferença significativa entre as crianças negras e brancas para a cobertura completa até os 24 meses de vida (RP = 0,21; IC_95_% 0,04;0,90), considerando-se as doses oportunas (Tabela 3).

## DISCUSSÃO

O estudo encontrou menor cobertura, considerando-se as doses oportunas para as vacinas de múltiplas doses e do esquema vacinal básico completo (sem a vacina de febre amarela), entre crianças negras, na coorte de nascidos entre 2017 e 2018 na cidade de Natal, evidenciando que as desigualdades raciais representaram barreiras à completude do calendário de vacinação até os 24 meses de vida da criança.

Os achados deste estudo mostram um perfil da vacinação no Brasil no qual os estratos populacionais de menor condição socioeconômica possuem pior oportunidade de vacinação. As crianças negras tiveram prevalência de cobertura vacinal completa oportuna 79% menor, quando comparadas às crianças brancas.

Em contraponto, estudo realizado na cidade de Pelotas/RS, em 2015, mostrou que o grupo com melhores condições socioeconômicas apresentou maior risco de incompletude vacinal;^18^ no inquérito de base populacional, realizado nas capitais brasileiras em 2012, a raça/cor da pele da criança não foi associada com a vacinação incompleta.^19^

Corroborando os achados do presente estudo, um inquérito domiciliar realizado em São Luís/MA, em 2006, verificou que, mesmo após ajuste para fatores socioeconômicos, as diferenças nas coberturas segundo a raça/cor da pele se mantiveram, ressaltando que crianças negras têm maiores dificuldades de serem vacinadas.^20^

Essa desigualdade injusta e evitável pode ser explicada pelo racismo estrutural que se manifesta no campo da saúde. Esse fenômeno pode se materializar através de preconceitos internalizados, estereótipos e sentimentos negativos vinculados a características raciais ou étnicas de um grupo; pela manifestação interpessoal de comportamentos e práticas discriminatórias, que excluem e inferiorizam esses grupos, concedendo-lhes atributos de menor valor ou considerando-os sem valor em relação a outros; e como componente estrutural, legitimado e praticado por organizações, políticas e normas, por meio de tratamento não equitativo, discriminatório, negligente, com desvantagem no acesso a benefícios e morosidade na implementação de ações e políticas que favoreceriam as suas vítimas.^21^

No presente estudo, a maior parte das crianças negras pertenciam às classes C e D, com mães de baixa escolaridade, e apresentaram maior hesitação vacinal. Todavia, não houve diferença significativa quando comparadas às crianças brancas analisadas no estudo. 

Em Londres, no Reino Unido, um estudo avaliou o efeito global da educação materna na vacinação infantil e apontou que há relação direta entre escolaridade materna e vacinação infantil, e que houve diferença significativa na cobertura vacinal de crianças filhas de mães alfabetizadas e não alfabetizadas, mostrando que o aumento do nível de escolaridade teve impacto positivo na adesão à vacinação.^22^ Mulheres mais escolarizadas tendem a procurar mais os serviços de saúde e estarem mais cientes na prevenção da imunização, em comparação com as mulheres sem escolaridade.^23^

Entre as famílias de crianças negras deste estudo, há maior percentual de hesitação vacinal, porém sem diferença estatística em comparação às crianças brancas. O fenômeno da hesitação pode estar associado à descrença nas vacinas, ao desabastecimento de vacinas nas unidades de saúde, à falta de conhecimento relacionado às vacinas e às doenças imunopreveníveis, e à dificuldade de acesso aos serviços de saúde.^4,24^

Ainda nesse o tema, um estudo^2^ cita que a falsa percepção de que não é mais preciso vacinar seus filhos, por acreditarem que as doenças desaparecem ao longo do tempo, pode levar os pais a hesitar em vacinar, e que esses fatores estão associados aos determinantes da cobertura vacinal.

Ademais, a propagação de notícias falsas sobre eventos adversos da vacina na infância também está presente em populações negras e com baixa escolaridade, dificultando ainda mais a completude do esquema vacinal.^1^

Em nosso estudo, verificou-se que, entre as crianças negras, houve maior proporção de aglomeração familiar, o que denota ser esse um marcador de piores condições socioeconômicas. Em outro estudo,^25^ verificou-se que as crianças dos agregados familiares mais pobres tinham 36% mais probabilidade de não serem imunizadas do que as crianças dos agregados familiares mais ricos. De acordo com outra pesquisa,^19^ devido à aglomeração familiar, há uma probabilidade de o responsável pela criança ter dificuldade para se deslocar até a unidade de saúde, por não ter tempo disponível para realizar a vacinação da criança, o que pode comprometer o esquema vacinal da criança.

Outro importante achado do presente estudo foi a baixa cobertura vacinal com doses oportunas entre as crianças negras. É importante destacar que, quando a maioria das crianças é vacinada de forma oportuna, os indivíduos não vacinados e aqueles em que houve falha vacinal também ficam protegidos. Nesse contexto, um indicador que merece destaque é o atraso vacinal, que é definido como o não recebimento da dose em tempo oportuno, pois seus resultados podem afetar a população vacinada, deixando-a desprotegida e interferindo na imunidade da população.^26,^ ^27^

A explicação para esse fato é que, tradicionalmente, a porção mais favorecida da população recebe mais e melhores intervenções em saúde, resultando em maior cobertura vacinal nesse grupo. A cobertura vacinal está diminuindo com os anos em países de alta renda, o que indica que fatores que favorecem o acesso e a utilização de serviços podem estar convergindo para o fenômeno da hesitação vacinal. Todavia, há sinais de inversão desse padrão em alguns países de baixa e média renda, onde a cobertura está aumentando entre os mais pobres e se reduzindo entre os mais ricos.^8,19,28^

Esse achado específico deste estudo mostra que a desigualdade na cobertura vacinal segue o padrão esperado de maior cobertura entre os de melhor condição socioeconômica. Entretanto, já há sinais de inversão desse padrão, já que não foram encontradas diferenças significativas nas coberturas vacinais entre negros e brancos para a maioria das coberturas analisadas.

Identificar essas desvantagens segundo a raça/cor da pele ainda vem preencher uma lacuna de conhecimento no país. As desigualdades raciais nos indicadores de saúde têm contribuído consideravelmente no aumento das disparidades sociais.^1,11,20,25^

A Organização Mundial da Saúde (OMS) compreende a importância de pesquisas relacionadas à vacinação, visando à redução da mortalidade infantil, e destaca a relevância de dados de alta qualidade para o monitoramento, prevenção e criação de estratégias voltadas à melhor distribuição e administração de vacinas, além do suporte e apoio às famílias das crianças.^29^

Conforme disposto no art. 14 do Estatuto da Criança e do Adolescente, “é obrigatória a vacinação das crianças nos casos recomendados pelas autoridades sanitárias”. Ademais, é dever da família, da sociedade e do Estado assegurar prioritariamente a efetivação dos direitos referentes à vida e à saúde da criança e do adolescente.^30^

De modo geral, destacam-se como pontos fortes desta pesquisa, por ser um estudo de base populacional, o tamanho da amostra e o processo de amostragem, e a estratégia adotada para coleta das informações sobre a situação vacinal, como a extração direta do registro de vacinas e a fotografia das cadernetas de saúde da criança, o que pode inclusive reduzir possíveis vieses de aferição.

As limitações deste estudo relacionam-se aos possíveis vieses de informação, já que havia a possibilidade de o respondente da pesquisa não ser o pai ou responsável pela criança; e viés de participação, considerando-se que houve maior percentual de perda por não localização do endereço, por imóvel fechado e por recusa entre as famílias de classe econômica A (60,4%) e B (47,5%). Isso pode ter afetado o cálculo das coberturas vacinais de acordo com a raça/cor da pele, já que foi observada maior concentração de crianças de raça/cor da pele branca nesses estratos. Outra limitação diz respeito à junção das categorias de raça/cor da pele (pretos e pardos) em uma única categoria e a exclusão dos indivíduos amarelos e indígenas. Tendo em vista o baixo número de participantes dessas categorias na amostra, criar categorias únicas poderia causar maior variabilidade estatística e levar a intervalos de confiança mais amplos e menos precisos, ao se estimarem parâmetros populacionais. 

Nossos resultados refletem a necessidade de aperfeiçoamento do sistema de saúde brasileiro, com vistas à correção das iniquidades apontadas. Entre as ações de promoção e prevenção, destacamos que as ações educativas devem abordar informações sobre a importância da vacinação infantil; e a comunicação deverá ser contínua, em linguagem simples, consistente e culturalmente adequada.

Além da participação dos familiares, a rede de atenção à saúde da criança deverá garantir o acesso aos imunobiológicos, bem como equipamentos para conservação de sua qualidade; a capacitação de seus profissionais quanto à adequada administração de vacinas e às orientações de cuidados pós-vacinais e à observância ao cumprimento do plano de vacinação; além da oferta de oportunidades para a atualização vacinal dos suscetíveis, promovendo a proteção coletiva contra as doenças imunopreveníveis.

O fortalecimento das ações estratégicas do PNI poderá incrementar o acesso aos serviços de saúde, bem como a oferta e o uso desses serviços para a parcela da população que mais necessita, com vistas à diminuição das desigualdades raciais/sociais. Além disso, é necessário proceder a avaliações sobre a qualidade do cuidado ofertado e sobre o desempenho do sistema de saúde, a partir de quesitos como adequação, continuidade, aceitabilidade, efetividade, eficiência, segurança e respeito aos direitos dos indivíduos.
